# Living and leaving a life of coercion: a qualitative interview study of patients with anorexia nervosa and multiple involuntary treatment events

**DOI:** 10.1186/s40337-023-00765-4

**Published:** 2023-03-13

**Authors:** Benjamin Mac Donald, Sanna A. Gustafsson, Cynthia M. Bulik, Loa Clausen

**Affiliations:** 1 Department of Clinical Medicine, Aarhus University, Palle Juul Jensens-Boulevard 99, 8200 Aarhus, Denmark; 2 Department of Child and Adolescent Psychiatry, Aarhus University Hospital, Palle Juul-Jensens Boulevard 175, Entrance K, 8200 Aarhus, Denmark; 3 School of Law, Psychology and Social Work, Örebro University, Örebro, Sweden; 4 Department of Psychiatry, University of North Carolina at Chapel Hill, Chapel Hill, NC USA; 5 Department of Medical Epidemiology and Biostatistics, Karolinska Institutet, Stockholm, Sweden; 6 Department of Nutrition, University of North Carolina at Chapel Hill, Chapel Hill, NC USA

**Keywords:** Adverse effects, Coercion, Compulsory treatment, Eating disorders, Patient experiences

## Abstract

**Background:**

A small but significant group of patients with anorexia nervosa (AN) undergo multiple involuntary treatment (IT) events. To enhance our understanding of IT and potentially inform treatment, we explored experiences and perspectives on IT of these patients.

**Methods:**

We designed a qualitative semi-structured interview study and used reflexive thematic analysis. Participants were at least 18 years of age, had multiple past IT events (≥ 5) related to AN over a period of at least one month of which the last IT event happened within the preceding five years. Participants had no current IT, intellectual disability, acute psychosis, or severe developmental disorder. We adopted an inductive approach and constructed meaning-based themes.

**Results:**

We interviewed seven participants. The data portrayed a process of living and leaving a life of coercion with a timeline covering three broad themes: living with internal coercion, coercive treatment, and leaving coercion; and five subthemes: helping an internal battle, augmenting suffering, feeling trapped, a lasting imprint, and changing perspectives. We highlighted that patients with AN and multiple IT events usually experienced internal coercion from the AN prior to external coercion from the health care system. IT evoked significant negative affect when experienced, and often left an adverse imprint. Moreover, IT could help an internal battle against AN and perspectives on IT could change over time.

**Conclusions:**

Our study suggests that feeling internally coerced by AN itself sets the stage for IT. Clinicians should be conscious of the potential iatrogenic effects of IT, and reserve IT for potentially life-threatening situations.

## Abstract (summary)

Some patients with anorexia nervosa refuse treatment and receive multiple involuntary treatment events. To learn more about involuntary treatment and potentially inform treatment, we explored experiences and perspectives of patients with multiple involuntary treatment events. We conducted interviews with seven participants and used a qualitative approach, namely reflexive thematic analysis, to construct themes. The data portrayed a process of living and leaving a life of coercion. We highlighted that patients with anorexia nervosa and multiple involuntary treatment events experience a life of coercion—both internally from the illness itself and from involuntary treatment experiences. Involuntary treatment can help an internal battle against anorexia nervosa and perspectives of patients about involuntary treatment can change over time. Nevertheless, involuntary treatment can have a negative impact on patients, such as feelings of being hunted or assaulted. Wider awareness of potential negative effects of involuntary treatment is needed. Clinicians should be conscious of these potential negative effects and reserve IT for potentially life-threatening situations.

## Background

Patients with anorexia nervosa (AN) commonly refuse treatment [1] and may be subject to involuntary treatment (IT) procedures in inpatient settings [2]. IT is defined as governed by legislation differentiating it from informal pressure or "*suasion*" [3, p. 391]. IT of patients with AN may save lives [4] when it is used in life-threatening circumstances with the legal prerequisites being a serious mental disorder and constituting a danger to oneself or others [5]. Both legislation and practice are known to differ between countries and hospitals [5–8]. In Denmark, IT may be used when the patient resists treatment, refuses food, attempts to abscond, purges, demonstrates aggression towards staff, engages in self-harm, or refuses necessary medical treatment. Some IT measures, e.g., mechanical or physical restraint, include direct interaction between caretaker and patient, but are relatively time-limited interventions compared with involuntary admission and detention that require a patient to be or to remain admitted, sometimes for several weeks. Nevertheless, all IT measures in Denmark take place in the hospital and entail involuntary hospitalization per se [9].

A few studies on patient experiences and perspectives have highlighted IT in AN as a negative experience. Patients state, "*while coercion may 'save lives', it may also 'kill spirits' so to speak*" [10, p. 133] or "*'it felt like punishment'*" [11, p. 640]. Moreover, experiencing specific IT measures (nasogastric tube feeding, involuntary medication, sedative medication, and restraint) has been described negatively [﻿^12^, ^13^], for instance as anxiety inducing and as "*punishment*" ﻿[13, p. 357]. Hence, IT can be a double-edged sword. On one hand, when used with individuals who have severe illness and are refusing treatment it can save lives [﻿^14^] and lead to similar outcomes as in patients who engage in treatment voluntarily [15]. At the same time, IT carries possible negative effects on patients [16].

Studies reported that when patients resisted IT, including nasogastric tube feeding, IT escalated and they were subject to measures such as physical restraint or sedative medication [13, 17]. Patients possibly resisted IT to restore autonomy [13] or achieve a sense of control [11]. This process may have been strengthened by the "*anorexic identity*" of patients and may have led to learned helplessness, which has been suggested to represent a turning point and the beginning of treatment acceptance ﻿[13, p. 359]. The experience of IT may be positively influenced by a trusting relationship between the patient and staff [18, 19], patient-centered care, good communication, giving sufficient information, and giving patients a say in treatment decisions [13, 20–22].

Some reports suggest that patients with AN change their perspectives on IT over time and view IT as necessary and helpful in retrospect [10, 17, 18, 23]. Furthermore, a study found no association between involuntary admission and subsequently reported satisfaction with overall treatment, hypothesizing that "*it is likely that clinical improvement* [may] *provide a retrospective meaning to the measures*" [24, p. 32].

Studies have found that a small number of inpatients with AN received multiple IT events [25–28] and were thus significantly affected by IT. In fact, 10% of patients with AN and IT in Denmark had a mean 5-year rate of 221 IT events [25]. However, to the best of our knowledge, experiences and perspectives on IT have not previously been explored with an exclusive focus on patients with multiple IT events. In order to enhance our understanding of the experience of IT through patients’ eyes, potentially inform treatment, and aid prevention of IT, we explored experiences and perspectives on IT in patients with AN who had experienced multiple IT events.

## Methods

### Participants

Participants were recruited in Denmark according to the following criteria: at least 18 years of age (Danish legal age), multiple past IT events (≥ 5) related to AN over a period of at least one month (i.e., a single short-term involuntary admission of less than one month's duration would not fulfill this criterion, whereas five short-term involuntary admissions spanning more than one month in total would) of which the last IT event happened within the preceding five years. Exclusion criteria: current IT, intellectual disability, acute psychosis, or severe developmental disorder.

Recruitment, interviews, and follow-up contact were conducted by the primary author (BMD). BMD is a male clinical psychologist specialized in child and adolescent psychiatry with experience in inpatient treatment of patients with AN. He was not involved in treatment at the time of the interviews and had not been involved in the treatment of the included patients. BMD aspired to a role of a student in the researcher–participant relationship [29].

### Recruitment

Using flyers, home page posts, and Facebook posts, we recruited participants purposively from June 2020 to mid-October 2021 through specialized hospital units, selected specialised residential units, the Danish patient organization, and the Danish Society for Eating Disorders. Participants chose the setting for the physical interview, which was either at their place of residence or Aarhus University Hospital. None of the included participants dropped out. Recruitment ended after an inclusion period of one year with several invitations for participation.

### Data collection

The study procedure included an assessment phone call regarding participation criteria, an interview, and a telephone follow-up interview. Interviews and follow-up interviews were audio recorded, interviews were by telephone, in person, and occasionally in the presence of a supportive person. Information at the assessment telephone call was handwritten by BMD.

A semi-structured interview guide was developed and continuously adjusted. To ensure a common understanding, the interview guide presented a definition of IT that highlighted its initiation according to the law. A semi-structured follow-up interview guide was also developed to further explore the experiences and perspectives of patients that arose from the initial interviews.

Interviews lasted 77 min on average (range: 65–88 min) and follow-up interviews lasted 34 min on average (range: 23–43 min).

Data saturation was not a criterion as Braun and Clarke [30, p. 210] state "*new meanings* [and themes] *are always (theoretically) possible*". However, both consistent and nuanced themes were obtained from the interviews.

### Data analysis

The method used for data processing was reflexive thematic analysis adopting an inductive approach and constructing meaning-based themes [31–33]. We engaged in bracketing our preconceptions, e.g., that the participants would consider IT a problem, by continuously using a reflexivity audit trail to gain awareness of our role and influence on the study process, while at the same time regarding our influence on the study as inevitable [29, 34].

Interviews and follow-up interviews were transscribed without editing by a research assistant with a bachelor's degree in psychology. The data were analysed using NVivo 12 [35]. The transscribed interviews were in Danish and coding was done iteratively by BMD in English and discussed with LC and SG. The participants did not corroborate the themes. Consolidated criteria for reporting qualitative research (COREQ) guidelines were used [36].

## Results

### Participants

Seven adult female participants in their 20s and 30s were included. Four currently had AN or atypical AN. Six had been diagnosed with comorbid psychiatric diagnoses (personality disorders, depression, autism spectrum disorders, obsessive compulsive disorder, attention-deficit hyperactivity disorder, and schizotypal disorder). They each had from five to above 100 IT events. None of the participants experienced only involuntary admission or detention. Examples of IT events were involuntary admission, detention, mechanical restraint, physical restraint, nasogastric tube feeding, and constant observation, i.e., the constant presence of a professional.

### Themes

**Fig. 1:**
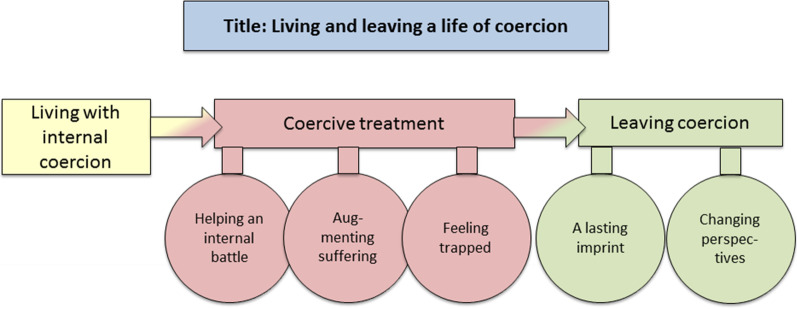
Themes and subthemes

The data portrayed a process of living and leaving a life of coercion with a timeline covering three broad themes; living with internal coercion, coercive treatment, and leaving coercion; and five subthemes; helping an internal battle, augmenting suffering, feeling trapped, a lasting imprint, and changing perspectives (see Fig. 1).

Patients' understandings of reasons for IT may be relevant to understand the source of their perspectives. They included low weight and a need for nutrition, resistance or lack of cooperation, compulsive exercise, a risk of absconding, and crying. Most participants described reasons for IT being influenced by organizational concerns such as lack of time to talk with patients, following a ”routine” practice, or a specialized residence pushing for IT. Similar concerns were also among reasons why IT continued, for instance a municipality not following the psychiatric hospital's recommendations about a specialized residence or staff's expectations based on previous admissions. IT could also continue because it was the only way for the participant to legitimize eating.

Overall, the participants' opinions about IT included a general consensus that IT is occasionally necessary. They elaborated on this, stating that sometimes it can be difficult to reach and reason with a patient with AN, although it is not necessarily too late at the time of IT. It was described that staff should not be too hesitant using IT in order to avoid further escalation or death. At the same time, participants described ambivalence about the use of IT and that it is ideal not to use it. They described that IT could, at least sometimes, have been avoided, and that it was occasionally used inappropriately in their own cases. Some participants found it difficult to establish a dialogue with staff. Some participants stressed the importance of being careful not to use IT routinely, or stated that IT could make everything worse. The participants' opinions on when IT was justified included to save lives and in case of danger to oneself or others. The data also yielded coercion as a very familiar phenomenon to patients not only imposed upon them by the health care system, but also prior to that, internally by AN itself. As IT also affected some of the participants after hospitalization, we suggested that patients' perspectives on IT should be outlined on a timeline including their lives before and after IT.

### Living with internal coercion

The theme "living with internal coercion" reflected that the patients' AN dictated what they should or should not do. Participant quotes are shown in Table 1.

**Table 1:** Participant quotes related to the first theme "Living with internal coercion"

"*I realize that it's myself who coerces me to like do the told things that the eating disorder … all that it involves, right? I feel it like … enormously tiring and hard and I feel it is … not at all as if I have a life. Not a good life, not very much joy and that is only because I myself live out the actions and do what the eating disorder like … yes, is about.*"
"*… one thing was that I didn't eat anything, but also was … didn't sleep very much and was always standing up and … had well, like exercised involuntarily or had compulsions about everything.*"
"*Um oh well … for instance … it* [AN] *has forced me to self-harm, um … it has forced me to run away quite a lot and it has forced me to walk, well, 14 km many times a day and such … there have just been so many … patterns and such things that it has forced me to do, where you really … I didn't want to self-harm, I didn't want to walk either because I was extremely tired. But like that … it has just forced me to that I had to.*"

Almost all participants described how AN itself created a state of internal coercion, forcing them to follow a regime of strict dieting, starvation, self-harm, and/or engaging in compulsive exercise. However, at the time of the IT, the fusion between the patients and AN was great with most of them experiencing AN as ego-syntonic or at least partially ego-syntonic.

### Coercive treatment

The second theme reflected perspectives on being coerced in treatment and included the subthemes "helping an internal battle", "augmenting suffering", and "feeling trapped". Participant quotes are shown in Table 2.

**Table 2:** Participant quotes related to the second theme "Coercive treatment"

**"Helping an internal battle" subtheme﻿**
"*And at other times then, um … well then it is close to somehow having been a help, because then I haven't had to have this eternal struggle in my head about what's right or wrong.*"
"*That it was the only way that it was for my anorexia to say to me, well … you cannot do anything. Now, it's okay that you get food because you don't have the power right now. So, it was really a struggle with myself; that I was expected to take some control, too; and that I should also go against the anorexia. And I just couldn't at that time. So yes, I think it made me a little dependent on having some involuntary treatment around me, because otherwise it wouldn't have been okay to eat at all.*"
"*It … was just like … the tube directly into the stomach and then … you don't have to taste anything, you don't have to smell anything, you don't have to like you know um, take a stand on anything, um. And that, I think, was somehow extremely relieving.*"
**﻿"Augmenting suffering" subtheme**
" *it is an assault whether it's been done few or many times.*"
"*Well, because it feels like an assault.*"
"*Um … the worst experiences they've definitely been this being strapped tight and then just lying alone.*"
**﻿"Feeling trapped" subtheme**
"*and it becomes something like an escape, here and now. It is as if it is such a primal instinct that emerges in you. You feel like a hunted animal and you cannot get your more human reasoning into it at all. You resort to that reptile brain, and just feel … you think of ways out and 'escape'. All the time you are hunted somehow*"
"*So um … she thought I should have a tube on top of this meal that I had already had almost the double of … and then I got really scared, um … so I, um … escaped down to the yard.*"
"*Well, for instance, I try to run past them somehow, and then you are just like hunted down to the other unit or the other end. Not 'hunted', but it feels that way. And then, for instance there is always a table down in shielded* [refers to the closed ward] *kind of like this one. And then, you know, then maybe I'm standing there, and then two of the staff walk that way and two that way. And then one is standing so you can't jump over the table. So, it like turns into a … hunt.*"
"*I'm close to thinking that it's worse this with there being seven people holding you tight for an hour than it is if you are* [mechanically] *restrained for an hour … because you just keep going on and on and on fighting back when there are so many people on top of you, um … at least I calm down faster, because I know that those straps, they do not give in, but in principle a human being could (laughs a little), um*"
"*like the consequence was that if you don't cooperate, um … then it's like yes, um … then you get it* [nasogastric tube feeding] *anyway, then you're just belt restrained.*"

#### Helping an internal battle

All participants expressed that they generally did not consider IT helpful at the time of IT. However, they sometimes saw the benefit at the time of IT and described the helpfulness of knowing that involuntary nasogastric tube feeding would be used if they did not comply with refeeding treatment. Some participants described that IT involving nasogastric tube feeding supported them in a battle in their minds against AN. That is, IT helped against an ongoing internal battle they had with their AN—it helped avoid the responsibility for getting nutrition, by placing the responsibility outside themselves, and some even described development of a reliance on nasogastric tube feeding and accompanying IT to receive nutrition.

#### Augmenting suffering

We found that the participants generally experienced IT as negative and that it added to their suffering. They used many negative words to describe IT, for instance abuse or assault, punishment, transgressive, unpleasant, fierce, and shameful. They described feeling powerless and not listened to or understood. IT was described as a loss of control with the elaboration that control was an important part of AN. IT also entailed a loss of meaning of life provided by using AN as a coping strategy, and the lack of any substitution could contribute to suicidality. Most participants experienced that IT created panic or anxiety, typically related to fear of nutrition or gaining weight.

Some of the participants also emphasized specific IT measures as negative. Constant observation was described as being transgressive; there was a description of constant observation being unbearable, although it was not considered to be IT at the time. Similarly, physical restraint was described as distressing and inhumane, while mechanical restraint was described as transgressive, degrading, worst, or difficult. Moreover, some participants experienced nasogastric tube feeding as unpleasant or scary.

#### Feeling trapped

Participants' descriptions of IT give a picture of feeling trapped or hunted, which resulted in resistance and attempts to escape. They also used other strategies such as yelling, crying, shutting the world out, kicking, spitting, self-harming, begging for help, and dissociating.

Some participants resisted IT in any way possible and resistance could lead to an escalation of IT, such as restraint after nasogastric tube feeding to prevent self-mutilation or purging behavior. Sometimes, IT could escalate to continuous nasogastric tube feeding while mechanically restrained. A few participants stated that they only resisted in the beginning of their course of treatment, possibly reflecting acceptance or learned helplessness.

### Leaving coercion

This theme reflected how the patients gained a new perspective and could reflect on the life-saving aspects of IT in retrospect, while at the same time still being affected by IT. The theme included the subthemes "a lasting imprint" and "changing perspectives". Participant quotes are shown in Table 3.

**Table 3:** Participant quotes related to the third theme "Leaving coercion"

**"A lasting imprint" subtheme**
"*it is some things that are still part of you, but it's something I'll never forget.*"
"*And the thing about you becoming so sensitive to the sounds around you, because then you just become like an animal that can hear okay this is it. I think that if you experience it once or twice then okay. But so many times, then it leaves some traces.*"
**"Changing perspectives" subtheme**
"*When you then … when you come more out on the other side, then you are able to see that what they did back then, at least some of it, has helped to you still being here*"
"*Hmm … I think now I'm able to look at it a little more objectively, um … because it's something else when you're exposed to it than when you look at it from the outside. Um, but from the outside then I would be able to rationalize that I was is situations where there was simply no other option than that.*"
"*So, I also think it's a bit about that I … that I … I have gotten this self-care, um … and dare to take* [use] *it and treat myself properly.*"
" *I'm not as destructive towards myself anymore, um, as I was back then. Because they gave me involuntary treatment, but that was also a way of punishing myself just like the eating disorder was, and the self-harm and all those things. It was somehow that I had to punish myself. And now, I don't punish myself.*"

#### A lasting imprint

The participants experienced that IT had a negative imprint on them, often for a long time. Most of the participants described effects bordering on trauma symptoms, such as dreaming about IT, fear of being touched, trying to forget the experiences, or trying to avoid particular IT measures including mechanical restraint, nasogastric tube feeding, and involuntary admission. Persistent attempts to avoid physical restraint could occur after having witnessed it. Moreover, IT as contributing to low mood was described, although infrequently.

#### Changing perspectives

The participants generally changed their understanding of IT and perceived it necessary and helpful in retrospect, and most of them described a change in their way of treating themselves during recovery from AN.

The participants described retrospectively viewing IT as necessary, unavoidable, meaningful, or helpful at least to some extent. The latter, though, not necessarily in ways that we imagine were intended by professionals. For instance, IT, among other aspects, could help a participant become motivated to avoid re-experiencing it. The expedient effect of IT in retrospect contrasts with the perception at the time of IT. In retrospect, all participants were able to reflect further on what staff aimed to achieve with IT, such as saving lives and ultimately wanting to aid their recovery.

Most of the participants described that their way of treating themselves had changed since the time of IT to include not being self-destructive, being able to prevent deterioration, not coercing themselves, not punishing themselves so much, and being more caring towards themselves. Only a few of the participants described that their way of treating themselves had not changed and that they had not recovered and were still very affected by AN.

## Discussion

To the best of our knowledge, the present study is the first study to thoroughly explore the experiences and perspectives on IT of patients with a history of multiple IT events. We portray a life of coercion that harbors the possibility of leaving coercion behind. The data covered three broad themes; living with internal coercion, coercive treatment, and leaving coercion.

The temporal dimension (Fig. 1) provides a framework for understanding the participants' experiences with IT, where the experiences covered by the first theme, living with internal coercion, helps explain subsequent experiences with IT. We construe the existence of a pre-existing internal coercion as almost all participants described an experience of internal coercion by the AN, as if AN drove them to engage in behaviors against their will. Hence, they described already being familiar with coercive strategies before experiencing IT, particularly coercing themselves to following their anorectic thoughts. Upon contact with the treatment system, they then encountered an external pressure to engage in treatment voluntarily. However, this was obviously not possible, because IT was initiated. In this way, IT occurs against the backdrop of coercive AN. Compulsivity related to AN has previously been described [37]. Moreover, at the time of IT, the fusion between the patient and AN was great, probably reflecting previous descriptions of AN as ego-syntonic [38].

We found it pivotal that participants felt trapped when they experienced IT. Descriptions of resistance and attempts to escape underpinned this feeling. Experiencing "*being confined*" has previously been described in involuntarily admitted psychiatric patients [39, p. 503]. Our understanding is that a collision between the internal coercive anorectic pressure, with which the participants tend to identify, and the external pressure from IT can produce the adverse experiences and intense negative emotional reactions, such as panic, anxiety, and feelings of being trapped or hunted that help fuel patients' resistance. They resist IT using different strategies, such as attempts to escape, in order to handle the experiences. Resistance in patients with AN undergoing IT, especially nasogastric tube feeding, has previously been described as leading to an escalation in IT [13, 17]. Similarly, our participants reported that their resistance tended to activate or escalate IT. In a way, the patient's and the system's rigidity get locked into a vicious cycle. However, one should not overlook the possible negative impact of the IT itself, i.e., IT is by definition treatment against the will of the person and, therefore, negatively experienced.

Although the participants mainly described adverse experiences and that IT added to their suffering, the findings suggest the existence of an ambivalent relationship with some participants acknowledging that IT helped them in an internal battle with AN—a battle they could have with their anorectic thoughts or perhaps their anorectic voice [40]. The "internal battle" with AN and the possibility that involuntary admission may alleviate the guilt patients can have about eating have been described by others [41, 42].

The participants described that experiencing multiple involuntary nasogastric tube feedings may lead to a reliance on this measure and accompanying IT, as it might bring relief from the internal battle with AN and exempt the patient from responsibility for her own treatment, thereby causing her to engage in a pattern of continued IT that should be avoided. Potential reliance on voluntarily administered nasogastric tube feeding has previously been described [43].

We found that IT often left a negative imprint that persisted long after the experiences. For some this negative imprint with trauma-related symptoms was concerning and this highlights the importance of carefully measured use of IT. This argument is substantiated by the other potential iatrogenic effects of IT, i.e., the added suffering from IT, the vicious cycle with reciprocal escalation of resistance and IT, the lack of a substitute coping strategy leading to suicidality as described, and the possible development of a reliance on nasogastric tube feeding and accompanying IT.

We also found that participants' perspectives on IT changed when they reflected on them post treatment. They gained an understanding of staff intentions and of the necessity, meaningfulness, and helpfulness of IT. The latter change in perspective where the participants came to see the positive potential of IT confirms previous findings [17, 18, 23, 24, p. 32]. Moreover, recovery seemed to influence the experiences of the participants. They described changes in their own way of treating themselves as the AN eased. They described new strategies that reduced or replaced the internal coercive AN including becoming more caring towards themselves. Self-compassion seems essential in the recovery process as either a means or result of the process of leaving a life coercion [44, 45].

The participants opined that IT was justified in order to save lives, in accordance with previous research, and in cases of danger to oneself or others [18]. Finally, the participants described several nuanced reasons for IT with some overlap in reasons why IT was initiated versus continued. The reasons presented by the participants expand on previous results from quantitative research [25]. Moreover, some of the perceived reasons would be difficult to justify according to Danish law [46], such as receiving IT due to crying, lack of time to talk with patients, or following a ”routine” practice. Such experiences can reflect either the use of unjustified IT or ineffective or insufficient debriefing with the patient after IT, both of which may worsen patients' perspectives on IT.

### Strengths and limitations

Some precautions are warranted. The findings are likely transferable to other patients with similar extensive experience with IT, but might not be comparable to patients with few IT events. As is best practice in reflexive thematic analysis [30–33], the researchers also influenced the findings, most obviously through the interview questions and interpretation of data.

With reference to sample size and data saturation, we consider the number of participants to be sufficient, because we were able to explore experiences and perspectives on IT of patients with multiple IT events and used the data to present a nuanced portrait of living and leaving a life of coercion [30]. Nevertheless, we acknowledge that the small sample size limits the transferability of the result.

Our study explored patients with multiple IT events and we did not narrow our focus to only a specific type of IT measure. As stated above, none of the participants experienced only involuntary admission or detention. Likewise, previous studies have shown that patients with AN who receive involuntary admission or detention often experience other IT measures as well [9, 25]. Hence, patients with multiple IT events most likely experience a mix of IT measures generating an overall imprint of IT and with specific measures having different individual connotations. Moreover, the IT may not always have been directly and solely related to AN. IT can be initiated for various reasons, as stated by the participants. Circumstances and psychiatric disorders other than AN alone, may have prompted some of the IT events experienced.

The study involves a risk of self-selection bias, but per se we cannot comment on those who chose not to participate. We had the impression that COVID-19 complicated recruitment. The participants gave the impression that sharing their experiences was important, did not appear afraid of reprisals, and seemed comfortable speaking freely during the interviews.

An unmeasured risk of memory bias exists. This was partially addressed by only including patients with the last IT event within the preceding five years. Furthermore, the interview guide involved focusing on specific IT events to reduce memory bias. The participants were prompted to talk about particular IT experiences, but as mentioned, they tended to talk about their general experience of IT.

We acknowledge the context dependence of the participants' experiences and that other conditions, such as relationships with staff, specific hospital wards, etc., may be important to the experience of IT. For instance, the importance of trusting patient–staff relationships in AN has previously been described [18]. It is also possible that there were conditions beyond the awareness of participants that affected their experiences of IT, such as cultural or legal conditions.

## Conclusions

To conclude, through the eyes of patients with multiple IT events, we portrayed a life of coercion that is primarily tormenting, but also a necessity in some life-threatening cases. While receiving IT measures, patients report feeling trapped or hunted, whereas in retrospect, the IT is generally understood as more meaningful. However, the participants highlight important factors for clinicians to consider as IT entails potential iatrogenic effects, such as augmentation of suffering and should be reserved for potentially life-threatening situations. IT measures may relieve patients from their experience of being internally coerced by AN, but the latter sets the stage for IT measures, and, when the patient has fused with the AN, the intensity of the AN and its treatment setting may result in escalation of IT and increased suffering and feelings of being trapped. Future research is warranted that explores the relational dynamic prior to initiation of IT, factors that prevent IT from being necessary, and appropriate debriefing approaches to help patients understand and appreciate its use and assuage feelings of being trapped or hunted. Staff experiences and perspectives on IT of patients with multiple IT events should also be further explored. Such research could contribute to our understanding of the dynamic of IT in this vulnerable treatment group.

## Data Availability

The interviews and NVivo 12 codes are not publicly available.

## Abbreviations

AN: Anorexia nervosa
COREQ: Consolidated criteria for reporting qualitative research
IT: Involuntary treatment
